# Regulation of S100A10 Gene Expression

**DOI:** 10.3390/biom11070974

**Published:** 2021-07-02

**Authors:** Aleksandra Głowacka, Paweł Bieganowski, Ewelina Jurewicz, Wiesława Leśniak, Tomasz Wilanowski, Anna Filipek

**Affiliations:** 1 Nencki Institute of Experimental Biology, Polish Academy of Sciences, 3 Pasteur Str., 02-093 Warsaw, Poland; al.glowacka@nencki.edu.pl (A.G.); e.jurewicz@nencki.edu.pl (E.J.); w.lesniak@nencki.edu.pl (W.L.); 2 Mossakowski Medical Research Institute, Polish Academy of Sciences, 5 Pawińskiego Str., 02-106 Warsaw, Poland; pawelb314@gmail.com; 3 Institute of Genetics and Biotechnology, Faculty of Biology, University of Warsaw, 1 Miecznikowa Str., 02-096 Warsaw, Poland

**Keywords:** cancer, gene expression, grainyhead-like 2, S100A10

## Abstract

S100A10, a member of the S100 family of Ca^2+^-binding proteins, is a widely distributed protein involved in many cellular and extracellular processes. The best recognized role of S100A10 is the regulation, via interaction with annexin A2, of plasminogen conversion to plasmin. Plasmin, together with other proteases, induces degradation of the extracellular matrix (ECM), which is an important step in tumor progression. Additionally, S100A10 interacts with 5-hydroxytryptamine 1B (5-HT1B) receptor, which influences neurotransmitter binding and, through that, depressive symptoms. Taking this into account, it is evident that *S100A10* expression in the cell should be under strict control. In this work, we summarize available literature data concerning the physiological stimuli and transcription factors that influence *S100A10* expression. We also present our original results showing for the first time regulation of *S100A10* expression by grainyhead-like 2 transcription factor (GRHL2). By applying in silico analysis, we have found two highly conserved GRHL2 binding sites in the 1st intron of the gene encoding S100A10 protein. Using chromatin immunoprecipitation (ChIP) and luciferase assays, we have shown that GRHL2 directly binds to these sites and that this DNA region can affect transcription of *S100A10*.

## 1. Introduction

The S100 protein family consists of over 20 low molecular weight (10–12 kDa), Ca^2+^-binding proteins expressed only in vertebrates [1]. In the human genome, genes encoding 17 members of the S100 family are clustered on chromosome 1 (1q21) [2]. Despite their shared localization, expression of S100 genes is not spatiotemporally synchronized and each gene has its own expression pattern [3,4]. S100 proteins bind Ca^2+^ through two “EF-hand” motifs. In a Ca^2+^-bound form, they interact with a variety of protein ligands which, in consequence, leads to regulation of numerous processes inside and outside the cell [5,6].

**Figure 1:**
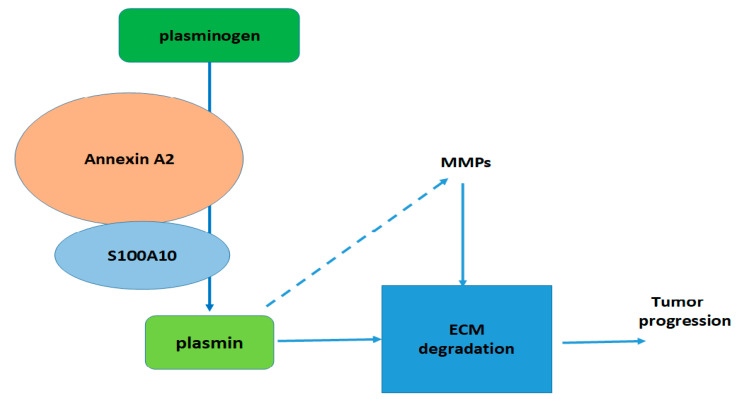
Role of S100A10 in tumor progression.

S100A10 is a unique member of the S100 protein family in that it does not bind Ca^2+^ and is insensitive to changes in Ca^2+^ concentration. Both “EF hand” motifs in S100A10 have amino acid substitutions that allow them to maintain a conformation, which resembles that of other S100 proteins in a Ca^2+^-bound form. This means that S100A10 is permanently locked in an active state [7]. Inside the cell, S100A10 forms a heterotetrameric complex with annexin A2, a protein that binds Ca^2+^ and phospholipids [8]. The annexin A2-S100A10 complex is involved in many membrane-associated processes, e.g., trafficking, fusion, microdomain or lipid raft organization and cytoskeleton-membrane binding. Additionally, this complex binds F-actin, which suggests that it is involved in cytoskeletal reorganization and regulation. Moreover, the annexin A2-S100A10 complex interacts with plasminogen and facilitates its conversion into plasmin, a serine protease that is responsible for fibrinolysis but can also activate metalloproteinases and degrade extracellular matrix (ECM) proteins [9]. Dysregulation of plasminogen conversion to plasmin plays an important role in the etiology of many cancer and non-cancer diseases. It is well known that degradation of extracellular matrix proteins, together with inflammation, promotes tumor growth, invasiveness, and metastasis (Figure 1). It is thus not surprising that an elevated level of S100A10 is a common feature of many cancers, such as squamous cell carcinoma, colon, lung, breast, or pancreatic cancer [10,11,12]. The role of S100A10 in other processes such as modulation of serotonin and other receptors, regulation of ion channel level and activity, or involvement in macrophage migration has been extensively reviewed [13,14]. It is only natural that expression of *S100A10* must be flexible enough to meet these functional demands. In this work, we present literature data concerning various factors that influence *S100A10* expression and our original results that show that the gene is regulated by GRHL2.

## 2. Regulation of *S100A10* Expression—Literature Data

The gene encoding S100A10 is highly inducible and its expression seems to be regulated by numerous external and internal factors. Studies performed by different research groups identified many physiological stimuli that promoted S100A10 expression, among them transforming growth factor-β (TGF-β), gonadotrophin (GD), epidermal growth factor (EGF), basic fibroblast growth factor (bFGF), interleukin 1β (IL-1β) [13], brain-derived neurotrophic factor (BDNF) [15], fibroblast growth factor 2 (FGF2) [16], and nitric oxide (NO) [17]. Theoretical analysis of the S100A10 promoter identified many potential regulatory sites, among them γIRE, GAS, and GRE, and consensus binding sequences for the following transcription factors: AP-1, Sp1, Sp2, NFκB, HIF1, ATF, and CTF-NF1 [13]. Over time, the binding of these and other transcription factors to their cognate binding sites within the regulatory regions of S100A10 has been verified experimentally, providing relevant information on the regulation of S100A10 expression. It was proposed, for example, based on the effects of siRNA-induced knockdown of several transcription factors, that c-Fos and c-Jun may act as activators, while CREM, Fosl2, STAT3, Sp1, and SRF may act as inhibitors, of S100A10 expression [16]. Indeed, the binding of the c-Fos/c-Jun dimer, which forms the AP-1 transcription factor, to the 1st exon of S100A10, was confirmed by chromatin immunoprecipitation (ChIP) and its stimulatory effect on expression was shown in a luciferase assay. Activation of S100A10 transcription by AP-1 is mediated by MAPK, PI3K, and JNK cascades in response to BDNF or FGF2 stimulation and constitutes one of the mechanisms of antidepressant response in which S100A10 plays a significant role [16]. Another group demonstrated the binding of Sp1, and probably also of Sp3, to a GC-box within the −96 to −70 segment of the S100A10 promoter using electrophoretic mobility shift assay (EMSA) [18]. It was shown that Sp1 upregulates S100A10 expression in response to nitrosative stress induced in motor neuron-like cell line, NSC34, by high NO concentration. Increased S100A10 level impairs the function of TASK1, a two-pore domain K+ channel, and contributes to degeneration of motor neurons, while silencing of S100A10 is neuroprotective [18].

The mechanism of the responsiveness of *S100A10* to inflammatory cues has also been studied. It was shown that, in response to interferon γ stimulation, STAT1 binds to two GAS sequences located at positions −1219 and −1090 of the gene promoter [19]. The effect of STAT1 binding on *S100A10* expression was assessed by the luciferase assay, which demonstrated that overexpression of wild-type STAT1 in two epithelial cell lines, BEAS-2B and HeLa, increased luciferase activity, while a phosphorylation deficient (Tyr 701) STAT1 mutant had no effect.

Chedeville et al. [20] observed increased *S100A10* expression following HIF1α upregulation in glioblastoma cells and samples from glioblastoma patients as compared with normal tissue [20]. Accordingly, the binding of HIF1α and HIF1β, but not HIF2α, to the 1st exon of *S100A10,* 103 bp downstream of the transcription start site, was shown by ChIP. This binding occurred following hypoxia or after treatment of glioblastoma cells with anti-cancer drugs [21]. The involvement of HIF1α links *S100A10* expression with hypoxic conditions characteristic for many cancer tissues.

It was also revealed that dexametasone-induced *S100A10* expression involves glucocorticoid receptor (GR) binding sites in the promoter sequence [22]. Three possible GRE sites (−223 to −241, −354 to −372, and −427 to −445) were identified in the *S100A10* promoter region; at least two of them were found to be functional by means of mutational analysis. The binding of GR to these sites was shown by ChIP after dexametasone stimulation of neuroblastoma SH-SY5Y cells [22].

The stimuli and transcription factors known to be involved in regulation of *S100A10* expression are listed in Table 1. Of note, in many cases, thanks to the research efforts described above, the whole signaling pathways, starting from the initial signal downstream to transcription factors that transduce the stimulatory cue into a higher transcription rate, have been revealed. Although most data concern a single activating stimulus and/or transcription factor, it is evident that in vivo these factors act in concert to modulate *S100A10* transcription according to cell’s needs.

## 3. Regulation of *S100A10* Expression by GRHL2 Transcription Factor—Original Data

### 3.1. Influence of GRHL2 on S100A10 Expression

GRHL2 is a member of the grainyhead-like transcription factor family [23]. GRHL2 regulates processes such as proliferation and differentiation [24] and thus its dysregulated expression is a common cause of malignant transformation. Interestingly, depending on the cancer type, the disease can arise due to either up- or downregulation of this transcription factor. This is because GRHL2 may act both as tumor suppressor, by inhibiting epithelial to mesenchymal transition, or as an oncogene, by inducing cell proliferation and hTERT expression. An increased level of GRHL2 was found in squamous cell carcinoma, gastric, liver, and colon cancer [25,26] and is usually associated with bad patient prognosis [24]. Increased expression of the gene encoding GRHL2 and, as mentioned above, of that encoding S100A10 is common for malignant transformation. Examples include hepatocellular carcinoma, non-small-cell lung cancer, colorectal cancer, and pancreatic ductal adenocarcinoma [11,26]. Thus, it was interesting to check whether GRHL2 transcription factor is involved in regulation of the gene encoding S100A10 protein. To show this, we used in silico and experimental approaches described in detail in Supplementary File (Materials and Methods).

**Figure 2:**
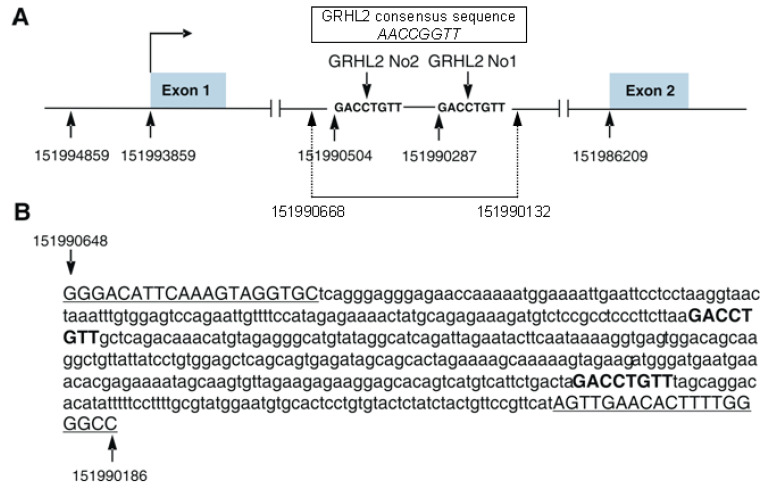
Schematic representation of the gene encoding S100A10 protein (**A**) and sequence of the intronic fragment amplified in ChIP (**B**). (**A**) Nucleotide sequence of two GRHL2 binding sites, GRHL2 No1 and GRHL2 No2. The 536 bp intronic fragment (151990668–151990132) cloned into pTA-Luc plasmid is indicated by dotted lines with arrowheads. (**B**) Nucleotide sequence of two GRHL2 binding sites (bold) and sequences covered by primers used for ChIP (underlined). Numbers are given according to GENEBANK NC_000001.11.

A theoretical analysis of putative GRHL2 transcription factor binding sites in the gene encoding human S100A10 protein (GENEBANK NC_000001.11 chr.1, GRCh38.p13 region complement 151982915-151994859) was performed using the MatInspector program (https://www.genomatix.de/solutions/genomatix-software-suite.html (accessed on 2 July 2021)). This program identified five potential, but only two highly conserved, binding sites for GRHL2 located in the 1st intron of *S100A10* (Figure 2A—GRHL2 No1 and GRHL2 No2 and Figure 2B—bold letters) [27]. These sites were only slightly divergent from the consensus 5′-AACCGGTT-3′ sequence. No sequence that might bind GRHL2 was found in the promoter region.

**Figure 3:**
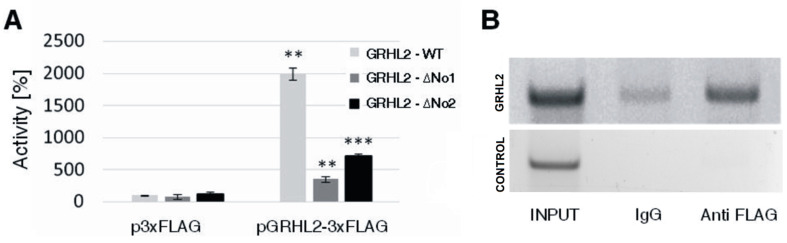
Influence of GRHL2 on *S100A10* expression and its binding to the *S100A10* intronic sequence. (**A**) Luciferase assay with plasmids containing the *S100A10* intronic sequence wild-type (GRHL2-WT) or lacking one of the GRHL2 binding sites (GRHL2-ΔNo1 or GRHL2-ΔNo2). Statistical analysis of results obtained from *n* = 3 experiments was performed with the use of Student’s *t*-test. Results are presented as means ± standard deviation. The level of statistical significance is indicated using ** p ≤ 0.01 or *** p ≤ 0.001. (**B**) A representative ChIP result—PCR products obtained with the use of primers encompassing both potential GRHL2 binding sites (upper panel) and primers that amplify a region of DNA not predicted to bind GRHL2 (lower panel).

To assess the influence of GRHL2 on *S100A10* transcription, a luciferase assay was employed. For that, a reporter plasmid (pTA-Luc-S100A10-GRHL2-WT) containing the luciferase gene and a 536 bp long intronic sequence (chr1:151 990 668-151 990 132), comprising the identified GRHL2 binding sites, was constructed as described in Supplementary File (Material and Methods and Table S1) and used in a Dual-Luciferase Reporter Assay System. For that, HEK293 (human embryonic kidney) cells were transfected with the pRL-SV40 reference plasmid, as an internal control, and with the pTA-Luc-S100A10-GRHL2-WT containing the examined sequence in the 1st intron of the *S100A10.* Cells were co-transfected with plasmid encoding GRHL2 with a FLAG tag (EX-W2222-M12-GRHL2-3xFLAG) or control plasmid (EX-NEG-M12-3xFLAG). Luciferase activity was assessed after 24 h using a Glomax 20/20 luminometer. As shown in Figure 3A, overexpression of GRHL2 led to an increase in luciferase activity, which indicates that this transcription factor regulates *S100A10* expression.

To confirm these results, each potential binding site for the GRHL2 transcription factor was subsequently deleted using pTA-Luc-S100A10-GRHL2-WT as a template in a PCR reaction with appropriate primers (Supplementary File, Table S1). The resulting products were named pTA-Luc-S100A10-GRHL2-ΔNo1 and pTA-Luc-S100A10-GRHL2-ΔNo2. The luciferase assay was performed as described above. As shown in Figure 3A, overexpression of GRHL2 in HEK293 cells transfected with plasmids containing the examined sequence lacking one of the GRHL2 binding sites led to a statistically significant decrease in luciferase activity.

To further assess the functionality of GRHL2 transcription binding sites in the 1st intron of *S100A10*, a ChIP assay was performed. For that HEK293 cells were transfected with plasmid EX-W2222-M12-GRHL2-3xFLAG and, 24 h later, were fixed with 1% formaldehyde, lysed, and sonicated into about 500 bp long fragments (Materials and Methods and Figure S1 in the Supplementary File). Overexpression of FLAG-tagged GRHL2 in the ChIP assay was necessary since available antibodies against GRHL2 are not specific and may recognize other transcription factors of this family. The lysate was incubated overnight with mouse IgG (control) or mouse monoclonal anti-FLAG antibody and the DNA-protein complexes were immunoprecipitated using Protein A/G Agarose. The immunoprecipitated DNA was used as a template for PCR reactions with primers flanking the two potential GRHL2 binding sites or primers flanking the region not predicted to bind GRHL2 (control) (Supplementary File, Table S1).

In accordance with an in silico analysis, which ranked the two GRHL2 sites as 22 and 37 top hits out of more than 255 hits (0.8 threshold) (Supplementary File, Table S2), a clear PCR product of an appropriate length was detected only in the sample containing the template immunoprecipitated with antibody against the FLAG tag and amplified with primers encompassing the two GRHL2 binding sites (Figure 3B, upper panel). No PCR product was visible in a control experiment (Figure 3B, lower panel). Thus, the results indicate that the GRHL2 transcription factor binds to its predicted sites within the 1st intron of *S100A10* and therefore may directly regulate expression of this gene.

### 3.2. Analysis of S100A10 mRNA in RC-124 Cells with Silenced GRHL2 Expression

To check the effect of the GRHL2 transcription factor on *S100A10* expression, we used kidney epithelial RC-124 cells with diminished level of this transcription factor (transfected with GRHL2 shRNA) [28]. The level of *GRHL2* mRNA in these cells and in control ones (transfected with scrambled shRNA) was analyzed using RT-qPCR. The TaqMan Fast Universal Master Mix with TaqMan Probes (ID: Hs02800695_m1 for *HPRT* and Hs00227745_m1 for *GRHL2*) was used and the reaction was performed in a 7900HT Fast Real-Time PCR System.

**Figure 4:**
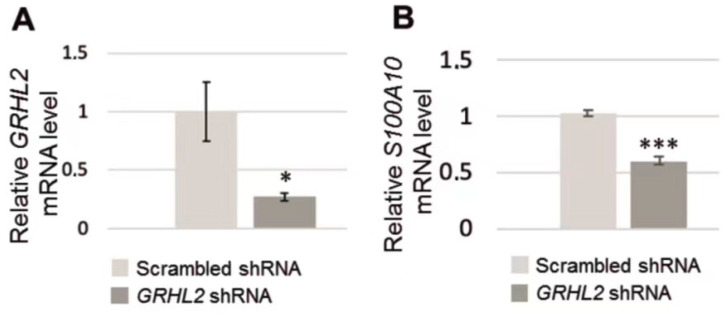
*S100A10* mRNA in RC-124 cells with silenced *GRHL2*. (**A**,**B**) RT-qPCR analysis showing the level of *GRHL2* mRNA and *S100A10* mRNA, respectively. (**A**,**B**): statistical analysis of results obtained from *n* = 3 experiments was performed with the use of Student’s *t*-test. Results are presented as means ± standard deviation. The level of statistical significance is indicated using * p ≤ 0.05 or *** p ≤ 0.001. Scrambled shRNA represents control cells while GRHL2 shRNA—cells with silenced *GRHL2* expression.

The obtained results were analyzed using the comparative ΔΔ*C*_t_ method and gene expression was normalized to the HPRT1 housekeeping gene. As can be seen in Figure 4A, indeed, the level of *GRHL2* mRNA in RC-124 with silenced *GRHL2* expression was diminished when compared to control cells.

*S100A10* mRNA level was then analyzed by RT-qPCR using the SYBRGreen system and appropriate primers (Supplementary File, Table S1). As it can be seen in Figure 4B, the level of *S100A10* mRNA in RC-124 cells with silenced *GRHL2* expression is lower than in control ones. In both cases, the differences were statistically significant.

In summary, by applying in silico analysis, we identified two highly conserved GRHL2 transcription factor binding sites in the 1st intron of the gene encoding the S100A10 protein. Such intronic location of the binding sites conforms to the results of ChIP-seq analyses, which show that GRHL2 binding occurs more frequently within intragenic and intronic regions than gene promoters [29,30]. Intronic GRHL2 binding sites were identified in *CDH1* (encoding E-catherin) and other genes [30,31]. The presence of regulatory sequences outside the promoter region is not uncommon [32]. For instance, it was found that 32–40% of transcription binding sites for HSF are located in introns [33]. Additionally, functional binding sequences for NFAT1 have been identified outside the gene promoter [34]. By performing chromatin immunoprecipitation (ChIP) and luciferase activity assays, we have found that GRHL2 directly binds to the intronic binding sites and that it is able to stimulate transcription of *S100A10*. Moreover, RT-qPCR showed that in cells with silenced *GRHL2* expression, *S100A10* mRNA level was lower than in control cells. Altogether, in this work, we show for the first time that GRHL2 transcription factor regulates expression of *S100A10*. Since both proteins are involved in proliferation/tumorigenesis/metastasis, their interrelated regulation might have some impact on cancer development and progression.

## 4. Conclusions

S100A10 is a member of the S100 Ca^2+^-binding protein family. S100A10 is involved in regulation of various processes, among them in plasmin production and regulation of the level of matrix metalloproteinases. Thus, in consequence, S100A10 has an effect on degradation of the extracellular matrix (ECM), which is an important step in tumorigenesis/metastasis.

Up to now, several reports have been published concerning regulation of *S100A10* expression. Based on all these data, it is evident that it can be influenced by many different stimuli, which, together with a range of transcription factors, ensure that the S100A10 protein level meets the demands of a given cell. Our work has identified yet another transcription factor, that is GRHL2, a protein which, among others, plays a role in tumorigenesis. Expression of GRHL2 is increased in many cancers [26] and the same applies to S100A10 [9]. Cancer types in which the expression of both GRHL2 and S100A10 is simultaneously increased include hepatocellular carcinoma, non-small-cell lung cancer, colorectal cancer, and pancreatic ductal adenocarcinoma [9,26]. In the above examples, the mechanism of GRHL2 action does not necessarily involve epithelial–mesenchymal transition. Taking into account the increased level of S100A10 and GRHL2 in many cancers, it is possible that regulation of *S100A10* expression by GRHL2 might have an impact on cancer progression and metastasis.

## Figures and Tables

**Table 1:** Stimuli and transcription factors which influence *S100A10* expression.

Stimulus	Transcription Factor	Reference
Transforming growth factor β (TGF β)	ND	[13]
Epidermal growth factor (EGF)	ND	[13]
Interleukin 1 β (IL-1 β)	ND	[13]
Brain-derived neurotrophic factor (BDNF); basic fibroblast growth factor 2 (FGF2)	c-Jun/c-Fos (AP-1)	[16]
Nitric oxide (NO)	Sp1, Sp3	[17,18]
Gonadotrophin (GD)	ND	[13,22]
Hypoxia, anti-cancer drugs	HIF1	[13,20,21]
Interferon γ	STAT1	[19,20]
ND	NFκB	[13]
ND	CTF-NF1	[13]
ND	GRHL2	Present work

ND—not determined.

## Supplementary Materials

The following are available online at https://www.mdpi.com/article/10.3390/biom11070974/s1, Materials and methods, Figure S1: Agarose gel showing the approximate length of the sheared chromatin used in ChIP, Table S1: Sequences of primers, Table S2: Putative transcription binding sites in S100A10 according to MatInspector.

## Abbreviations

BDNF	brain-derived neurotrophic factor
BSA	bovine serum albumin
ChIP	chromatin immunoprecipitation assay
DTT	dithiothreitol
EDTA	ethylenediaminetetraacetic acid
EGF	epidermal growth factor
FGF2	fibroblast growth factor 2
bFGF	basic fibroblast growth factor
GAS	interferon γ activation site
GD	gonadotrophin
GR	glucocorticoid receptor
GRE	glucocorticoid response element
GRHL2	grainyhead-like 2
hTERT	human telomerase reverse transcriptase
JNK	c-Jun N-terminal kinase
L-1β	interleukin 1β
IP_3_	1,4,5-trisphosphate inositol
γIRE	interferon γ response element
MAP	mitogen activated protein
TGF-β	transforming growth factor-β
