# Supplementary material for: Regulation of S100A10 Gene Expression
Source: Biomolecules. 2021 Jul 2;11(7):974. doi: 10.3390/biom11070974 (PMC8301800; doi:10.3390/biom11070974)
Supplement: Supplementary file 1 [file biomolecules-11-00974-s001.zip › biomolecules-1245542-supplementary.pdf]

Regulation of S100A10 gene expression

Aleksandra Głowacka[1], Paweł Bieganowski[2], Ewelina Jurewicz[1], Wiesława Leśniak[1], Tomasz Wilanowski[3#], Anna Filipek[1#]

[1]Nencki Institute of Experimental Biology, Polish Academy of Sciences, 3 Pasteur Str., 02-093 Warsaw, Poland

[2]Mossakowski Medical Research Institute, Polish Academy of Sciences, 5 Pawińskiego Str., 02-106, Warsaw, Poland

[3]University of Warsaw, Faculty of Biology, Institute of Genetics and Biotechnology, 1 Miecznikowa Str., 02-096 Warsaw, Poland

# Corresponding authors:

Anna Filipek
Nencki Institute of Experimental Biology PAS,
3 Pasteur Street, 02-093 Warsaw, Poland
Tel: +48-22-589 23 32; Fax: +48-22- 822 53 42
E-mail: a.filipek@nencki.edu.pl

Tomasz Wilanowski
University of Warsaw, Faculty of Biology, Institute of Genetics and Biotechnology,
1 Miecznikowa Street, 02-096 Warsaw, Poland
E-mail: t.wilanowski@biol.uw.edu.pl

Materials and methods

Theoretical analysis of the gene encoding S100A10 protein and plasmid construction

Theoretical analysis of putative GRHL2 transcription factor binding sites in the human *S100A10* (GENEBANK NC_000001.11 chr.1, GRCh38.p13 region complement 151982915 - 151994859) was performed using the MatInspector program (https://www.genomatix.de/solutions/genomatix-software-suite.html) set at a 0.8 threshold. For the luciferase assay a reporter plasmid (pTA-Luc S100A10-GRHL2-WT) containing the luciferase gene and a 536 bp intronic sequence (chr1:151 990 668 - 151 990 132), comprising the two identified GRHL2 binding sites was constructed in the following way. At first, the respective DNA fragment was amplified using primers S100A10-GRHL2-WT-F KpnI and S100A10-GRHL2-WT-R BamHI (Table S1) and then digested with KpnI and BamHI and cloned upstream of the minimal TA promoter into the pTA-Luc plasmid (Clontech) digested with the same enzymes. Each potential binding site for the GRHL2 transcription factor was subsequently

deleted and the resulting products were named pTA-Luc-S100A10-GRHL2-ΔNo1 and pTA-Luc-S100A10-GRHL2-ΔNo2. To create plasmids carrying these deletions, the pTA-Luc-S100A10-GRHL2-WT plasmid was used as a template in a PCR reaction with primers S100A10-GRHL2-ΔNo1-F and S100A10-GRHL2-ΔNo1-R or S100A10-GRHL2-ΔNo2-F and S100A10-GRHL2-ΔNo2-R (Table S1). The products of the reaction were gel-purified and subjected to a SLIC cloning procedure [1].

Cell culture and transfection

HEK293 (Human Embryonic Kidney) cells (Sigma-Aldrich) were cultured in DMEM supplemented with 10% FBS (Gibco), 40 mM sodium bicarbonate (Sigma-Aldrich), 100 µg/ml streptomycin and 100 U/ml penicillin (both from Sigma-Aldrich) in 5% CO_2 at 37ºC. Medium was changed every 2-3 days and the cells were passaged when confluent. To obtain transient overexpression of GRHL2 for chromatin immunoprecipitation (ChIP) experiments, HEK293 cells were transfected with EX-W2222-M12 plasmid encoding GRHL2-3xFLAG (GeneCopoeia) using Lipofectamine2000 (Life Technologies) according to the manufacturer's protocol. Cells were collected 24h after transfection.

RC-124 cells (human non-tumorigenic kidney cells) stably transfected with GRHL2-shRNA and RC-124 control cells transfected with scrambled shRNA were obtained earlier as described in [2]. Cells were cultured in McCoy's 5A (Biowest) with 10% FBS, 100 µg/ml streptomycin and 100 U/ml penicillin. Cultures were maintained in the presence of 5% CO_2 at 37°C. Medium was changed every 2–3 days and cells were passaged when confluent.

Luciferase assay

To check the influence of GRHL2 on *S100A10* expression the Dual-Luciferase Reporter Assay System (Promega) was applied. HEK293 cells seeded on 24-well plates were co-transfected with the pRL-SV40 reference plasmid (Promega) as an internal control and with plasmids containing the 536 bp long *S100A10* intronic sequence cloned upstream of the firefly luciferase gene. The sequence was either wild type (pTA-Luc-S100A10-GRHL2-WT) or lacked a potential GRHL2 binding site (pTA-Luc-S100A10-GRHL2-ΔNo1 and pTA-Luc-S100A10-GRHL2-ΔNo2). Cells were co-transfected with the EX-W2222-M12-GRHL2-3xFLAG (GeneCopoeia) or EX-NEG-M12-3xFLAG (control) (GeneCopoeia) plasmid. Luciferase activity was assessed after 24h using a Glomax 20/20 luminometer (Promega).

Chromatin immunoprecipitation (ChIP) assay

To perform the chromatin immunoprecipitation (ChIP) experiment HEK293 cells were transfected with plasmid: EX-W2222-M12-GRHL2-3xFLAG or EX-W2222-M12-3xFLAG (control) purchased from GeneCopoeia. 24 h later cells were fixed with formaldehyde added to the medium up to 1% final concentration, lysed, sonicated for 10 min (10 cycles: 30 s sonication, 30 s off) using Bioruptor Plus (Diagenode). The approximate length of the sheared chromatin has been examined by standard agarose electrophoresis at various times (5 - 30 min) of sonication and proved to be close to 500 bp (Figure S1).

The lysate was incubated overnight with mouse IgG (control) or mouse monoclonal anti-FLAG antibody (both from Sigma-Aldrich) and the DNA-protein complexes were immunoprecipitated using Protein A/G Agarose (Sigma-Aldrich). The immunoprecipitated DNA was used as a template for PCR reactions with primers GRHL2-F and GRHL2-R flanking the two potential GRHL2 binding sites and primers Control-F and Control-R that amplify a region of *S100A10* not predicted to bind GRHL2. The PCR program included initial incubation for 3 min at 95°C and then 35 cycles of 30 s at 95°C, 30 s at 47°C and 1 min at 72°C. PCR products were separated on 1.5% agarose gel.

Reverse transcription-quantitative PCR (RT-qPCR)

To confirm silencing of the gene encoding GRHL2 transcription factor in the RC-124 cell line (transfected with GRHL2 shRNA), total RNA was extracted from these cells and from control RC-124 cells (transfected with scrambled shRNA) using the ExtractMe Total RNA Kit (Blirt). Next, 1 µg of the RNA was reverse-transcribed to cDNA with the use of MMLV1 reverse transcriptase (Sigma-Aldrich) according to the manufacturer's protocol. The level of *GRHL2* mRNA was analyzed using TaqMan Fast Universal Master Mix (Applied Biosystems) with TaqMan Probes (ID: Hs02800695_m1 for *HPRT* and Hs00227745_m1 for *GRHL2*).

In order to assess the influence of GRHL2 transcription factor on *S100A10* mRNA level, total RNA was isolated from RC-124 cells with stably silenced *GRHL2* using the RNeasy Mini Kit (Qiagen) and 1 µg of the RNA was transcribed to cDNA as described above. *S100A10* mRNA level was then analyzed by RT-qPCR using the SYBRGreen system. The primers used were the following: S100A10-F and S100A10-R, and HPRT1-F and HPRT1-R.

RT-qPCR was performed in a 7900HT Fast Real-Time PCR System (Applied Biosystems). The obtained results were analyzed using the comparative $\Delta\Delta C_t$ method and gene expression was normalized to the HPRT1 housekeeping gene.

Statistical analysis

Statistical analysis of data obtained in all experiments was performed with the use of Student's *t*-test. Results of at least three independent experiments are presented as means ± standard deviation. The level of statistical significance is indicated using $*p \leq 0.05$, $**p \leq 0.01$ or $***p \leq 0.001$.

Table S1. Sequences of primers.

**Primers used in plasmid construction**	
S100A10-GRHL2-WT-F KpnI	5'-GCACCAGGTACCTCATGCCTAACACCATGTC-3'
S100A10-GRHL2-WT-R BamHI	5'-CGTCTGGATCCTTGTAGCTCAGAGCTCAGG-3'
S100A10-GRHL2-ΔNo1-F	5'-TAGCAGGACACATATTTTCCTTTTGC-3'
S100A10-GRHL2-ΔNo1-R	5'-AAAATATGTGTCCTGCTATAGTCAGAATGACATGACTGTG-3'
S100A10-GRHL2-ΔNo2-F	5'-TTAAGAAGGGAGGCGGAGACAT-3'
S100A10- GRHL2-ΔNo2-R	5'-CTCCGCCTCCCTTCTTAAGCTCAGACAAACATGTAGAGG-3'
**Primers used in ChIP**	
GRHL2-F	5'-GGGACATTCAAAGTAGGTGC-3'
GRHL2-R	5'-GGCCCCAAAAGTGTTCAACT-3'
Control-F	5'-AGCTTAAATCTCAGGCAGAC-3'
Control-R	5'-TCTCTTGGCTAGAGGATGAA-3'
**Primers used in RT-qPCR**	
S100A10-F	5'-GGCTACTTAACAAAGGAGGACC-3'
S100A10-R	5'-GAGGCCCGCAATTAGGGAAA-3'
HPRT1-F	5'-ACCAGTCAACAGGGGACATA-3'

HPRT1-R	5'-CTTCGTGGGGTCCTTTTCACC-3'

Figure S1. Agarose gel showing the approximate length of the sheared chromatin used in ChIP.

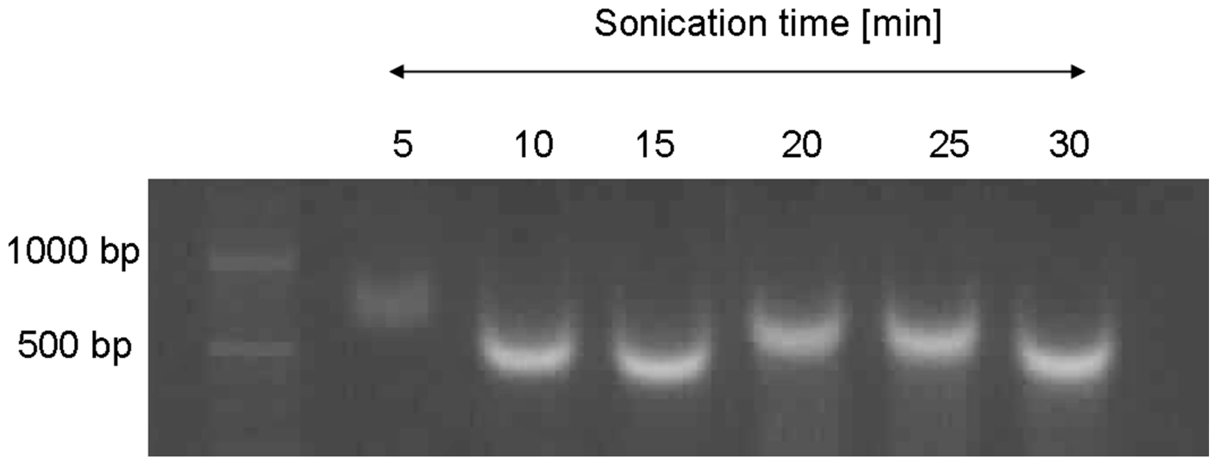

Table S2.

Putative transcription binding sites in *S100A10* according to MatInspector.

Matrix ID	Name	Score	Relative score	Sequence ID	Start	End	Strand	Predicted sequence
MA0035.4	GATA1	13,729	0,9784227	S100A10	187	197	-	ttctaatctga
MA0809.2	TEAD4	14,3618	0,9641916	S100A10	398	409	-	gcacattccata
MA1121.1	TEAD2	14,4116	0,9580448	S100A10	397	409	-	gcacattccatac
MA0508.3	PRDM1	13,1531	0,9485031	S100A10	114	124	-	atctttctctg
MA0079.3	SP1	12,7041	0,9409729	S100A10	126	136	+	tctccgcctcc
MA1585.1	ZKSCAN1	12,581	0,9334626	S100A10	10	19	+	aaagtaggtg
MA0744.2	SCRT2	15,6372	0,9290302	S100A10	142	157	-	tgagcaacaggtctta
MA0599.1	KLF5	9,7411	0,9271733	S100A10	23	32	-	ctccctccct
MA0090.3	TEAD1	13,4552	0,9260518	S100A10	397	409	-	gcacattccatac
MA0497.1	MEF2C	12,845	0,9059142	S100A10	32	46	+	gaaccaaaaatggaa
MA0479.1	FOXH1	9,37408	0,9044962	S100A10	415	425	-	tagagtacaca
MA1522.1	MAZ	10,0846	0,9015073	S100A10	128	138	+	tccgcctccct
MA1579.1	ZBTB26	11,1858	0,9009072	S100A10	80	94	+	gagtccagaattgtt
MA0465.2	CDX2	11,5472	0,8971283	S100A10	200	211	+	cttcaataaaag

MA1108.2	MXI1	7,03377	0,8954662	S100A10	376	385	+	gacacatatt
MA0769.2	TCF7	9,70378	0,8947387	S100A10	5	15	-	tactttgaatg
MA1645.1	NKX2-2	10,9934	0,8940783	S100A10	209	222	-	tgtccactcacctt
MA0743.2	SCRT1	13,3384	0,8923548	S100A10	142	157	-	tgagcaacaggtctta
MA0079.3	SP1	8,72447	0,8909046	S100A10	22	32	-	ctccctccctg
MA0516.1	SP2	12,1439	0,8888383	S100A10	126	140	+	tctccgcctcccttc
MA0083.2	SRF	14,1046	0,8885411	S100A10	30	47	+	gagaaccaaaaatggaaa
MA1105.2	GRHL2	11,4162	0,8876184	S100A10	360	371	-	taaacaggtcta
MA1683.1	FOXA3	9,63796	0,8875003	S100A10	413	423	-	gagtacacagg
MA0018.4	CREB1	10,9492	0,8868482	S100A10	342	354	+	cagtcatgtcatt
MA1108.2	MXI1	6,48412	0,8851957	S100A10	173	182	-	tatacatgcc
MA1108.2	MXI1	6,48174	0,8851512	S100A10	162	171	-	tctacatgtt
MA1508.1	IKZF1	11,0794	0,8850826	S100A10	385	396	-	gcaaaaggaaaa
MA1104.2	GATA6	11,1072	0,8849629	S100A10	187	199	-	tattctaatctga
MA0047.3	FOXA2	9,46208	0,8848897	S100A10	413	423	-	gagtacacagg
MA0036.3	GATA2	8,84809	0,8848845	S100A10	187	197	-	ttctaatctga
MA0599.1	KLF5	6,34072	0,8839158	S100A10	126	135	+	tctccgcctc
MA1103.2	FOXK2	9,48441	0,8818727	S100A10	413	423	-	gagtacacagg
MA0901.2	HOXB13	11,7255	0,8816079	S100A10	200	213	+	cttcaataaaaggt
MA1655.1	ZNF341	10,4003	0,8809131	S100A10	225	236	-	aataacagcctt
MA0024.2	E2F1	7,44529	0,8800341	S100A10	23	33	+	agggagggaga
MA0479.1	FOXH1	7,60655	0,8796315	S100A10	76	86	-	tggactccaca
MA1105.2	GRHL2	10,8306	0,8758423	S100A10	143	154	-	gcaacaggtctt
MA0593.1	FOXP2	8,94845	0,8746824	S100A10	89	99	-	tggaaaacaat
MA0597.1	THAP1	6,75558	0,8743871	S100A10	21	29	-	cctccctga
MA0052.4	MEF2A	12,2782	0,8741616	S100A10	33	47	+	aaccaaaaatggaaa
MA0037.2	GATA3	6,5876	0,8726537	S100A10	189	196	+	agattaga
MA0063.2	NKX2-5	8,27371	0,8700805	S100A10	3	13	+	gacattcaaag
MA0481.3	FOXP1	8,69208	0,8699558	S100A10	413	423	-	gagtacacagg
MA0482.2	GATA4	9,82648	0,8695286	S100A10	328	339	-	ctccttctcttc
MA1115.1	POU5F1	9,12918	0,869269	S100A10	39	49	+	aaatggaaaat
MA0597.1	THAP1	6,55651	0,8682948	S100A10	169	177	-	atgccctct
MA0528.2	ZNF263	10,1739	0,8676012	S100A10	129	140	-	gaagggaggcgg
MA1112.2	NR4A1	8,7838	0,8672427	S100A10	206	217	+	taaaaggtgagt
MA0144.2	STAT3	5,68154	0,866384	S100A10	111	121	+	atgcagagaaa
MA0471.2	E2F6	9,98714	0,8663148	S100A10	22	34	+	cagggagggagaa

MA0508.3	PRDM1	8,85474	0,8659253	S100A10	329	339	-	ctccttctctt
MA1648.1	TCF12(var.2)	8,27862	0,8648617	S100A10	11	21	-	agcacctactt
MA0508.3	PRDM1	8,79194	0,8647189	S100A10	255	265	-	tgctatctcac
MA0468.1	DUX4	5,29126	0,863296	S100A10	189	199	-	tattctaatct
MA0063.2	NKX2-5	7,95788	0,8629824	S100A10	210	220	-	tccactcacct
MA0522.3	TCF3	7,90104	0,862703	S100A10	11	21	-	agcacctactt
MA1108.2	MXI1	5,24076	0,8619624	S100A10	401	410	-	tgcacattcc
MA0470.1	E2F4	7,37494	0,8616004	S100A10	24	34	+	gggagggagaa
MA1522.1	MAZ	7,18289	0,8592381	S100A10	23	33	-	tctccctccct
MA1593.1	ZNF317	10,1579	0,8591183	S100A10	424	435	-	gaacagtagata
MA0488.1	JUN	6,33666	0,8567332	S100A10	341	353	+	acagtcatgtcat
MA1124.1	ZNF24	13,0632	0,8566149	S100A10	294	306	-	gtttcattcatcc
MA0510.1	RFX5	7,20723	0,8563502	S100A10	147	161	-	tgtctgagcaacagg
MA1115.1	POU5F1	8,49569	0,8552118	S100A10	4	14	+	acattcaaagt
MA0593.1	FOXP2	7,79825	0,8548699	S100A10	413	423	-	gagtacacagg
MA0468.1	DUX4	4,36099	0,8531696	S100A10	46	56	-	gaattcaattt
MA0596.1	SREBF2	9,01817	0,85299	S100A10	291	300	+	atgggatgaa
MA0037.2	GATA3	5,28073	0,8520906	S100A10	330	337	+	agagaagg
MA0599.1	KLF5	3,82933	0,8519673	S100A10	129	138	+	ccgcctccct
MA0037.2	GATA3	5,22756	0,8512538	S100A10	101	108	+	agagaaaa
MA0492.1	JUND(var.2)	5,03246	0,8493087	S100A10	340	354	+	cacagtcatgtcatt
MA1103.2	FOXK2	8,10085	0,849142	S100A10	364	374	-	tgctaaacagg
MA1108.2	MXI1	4,53932	0,8488555	S100A10	160	169	+	caaacatgta
MA0100.3	MYB	5,67086	0,8487535	S100A10	425	434	+	atctactgtt
MA0093.3	USF1	9,606	0,8477793	S100A10	342	355	+	cagtcatgtcattc
MA0492.1	JUND(var.2)	4,7765	0,8466982	S100A10	343	357	-	cagaatgacatgact
MA0258.2	ESR2	8,84369	0,8465998	S100A10	347	361	+	atgtcattctgacta
MA1585.1	ZKSCAN1	7,3366	0,8464386	S100A10	424	433	-	acagtagata
MA1115.1	POU5F1	8,09152	0,846243	S100A10	109	119	+	ctatgcagaga
MA1117.1	RELB	8,5175	0,8461247	S100A10	51	61	+	gaattcctcct
MA0106.2	TP53	10,8482	0,8460489	S100A10	174	188	+	gcatgtataggcatc
MA0852.2	FOXK1	9,33871	0,8447249	S100A10	411	424	-	agagtacacaggag
MA0143.4	SOX2	7,23111	0,8445699	S100A10	36	46	+	caaaaatggaa
MA0809.2	TEAD4	8,15872	0,8445165	S100A10	2	13	+	ggacattcaaag
MA0510.1	RFX5	6,13985	0,8441806	S100A10	14	28	-	ctccctgagcaccta
MA0037.2	GATA3	4,6395	0,842001	S100A10	271	278	+	agaaaagc

MA0095.2	YY1	8,05071	0,8416829	S100A10	37	48	+	aaaaatggaaaa
MA0060.3	NFYA	7,60742	0,8415331	S100A10	211	221	-	gtccactcacc
MA1644.1	NFYC	8,08342	0,8413562	S100A10	211	221	-	gtccactcacc
MA0599.1	KLF5	2,99184	0,8413133	S100A10	211	220	-	tccactcacc
MA0712.2	OTX2	6,79562	0,8410269	S100A10	186	197	+	atcagattagaa
MA0103.3	ZEB1	5,59228	0,8403021	S100A10	11	21	-	agcacctactt
MA0526.3	USF2	9,25138	0,8401121	S100A10	342	355	+	cagtcatgtcattc
MA0712.2	OTX2	6,7002	0,839127	S100A10	291	302	+	atgggatgaatg
MA1647.1	PRDM4	7,60199	0,8384995	S100A10	300	310	-	tcgtgtttcat
MA1111.1	NR2F2	7,61342	0,8381636	S100A10	207	217	+	aaaaggtgagt
MA0482.2	GATA4	8,30656	0,8379624	S100A10	187	198	-	attctaatctga
MA0597.1	THAP1	5,52713	0,8367908	S100A10	132	140	+	cctcccttc
MA0509.2	RFX1	9,44904	0,8361894	S100A10	148	161	+	ctgttgctcagaca
MA1118.1	SIX1	8,85203	0,8360971	S100A10	302	312	+	gaaacacgaga
MA0596.1	SREBF2	8,25388	0,8351408	S100A10	344	353	-	atgacatgac
MA1642.1	NEUROG2(var.2)	7,01277	0,8349767	S100A10	284	296	+	gtagaagatggga
MA0508.3	PRDM1	7,2112	0,8343503	S100A10	307	317	-	tattttctcgt
MA1657.1	ZNF652	8,98111	0,8338624	S100A10	318	329	+	gcaagtgttaga
MA0100.3	MYB	4,93658	0,8331198	S100A10	440	449	-	ttcaactatg
MA1125.1	ZNF384	5,95587	0,8329432	S100A10	200	211	+	cttcaataaaag
MA0489.1	JUN(var.2)	5,2845	0,8329364	S100A10	295	308	+	gatgaatgaaacac
MA1120.1	SOX13	6,74531	0,8327961	S100A10	36	46	+	caaaaatggaa
MA1632.1	ATF2	7,15405	0,8316645	S100A10	342	354	+	cagtcatgtcatt
MA0007.2	AR	6,61711	0,8313478	S100A10	377	391	-	aggaaaaatatgtgt
MA0593.1	FOXP2	6,41979	0,8311257	S100A10	364	374	-	tgctaaacagg
MA0764.2	ETV4	4,93141	0,8307986	S100A10	51	60	-	ggaggaattc
MA0037.2	GATA3	3,92068	0,8306907	S100A10	115	122	+	agagaaag
MA0037.2	GATA3	3,92068	0,8306907	S100A10	421	428	-	agatagag
MA0079.3	SP1	3,93537	0,8306521	S100A10	129	139	+	ccgcctccctt
MA1638.1	HAND2	3,64126	0,8302732	S100A10	118	127	+	gaaagatgtc
MA0100.3	MYB	4,79922	0,8301952	S100A10	317	326	+	agcaagtgtt
MA0258.2	ESR2	7,59541	0,8301092	S100A10	347	361	-	tagtcagaatgacat
MA1115.1	POU5F1	7,33417	0,8294372	S100A10	390	400	-	atacgcaaaag
MA0507.1	POU2F2	6,80292	0,8292878	S100A10	388	400	+	tccttttgcgtat
MA1120.1	SOX13	6,58729	0,8290896	S100A10	106	116	+	aaactatgcag
MA0627.2	POU2F3	7,6296	0,8286898	S100A10	3	15	+	gacattcaaagta

MA1683.1	FOXA3	7,06382	0,8285044	S100A10	364	374	-	tgctaaacagg
MA1631.1	ASCL1(var.2)	7,39739	0,8283739	S100A10	10	22	-	gagcacctacttt
MA0597.1	THAP1	5,24629	0,8281956	S100A10	25	33	-	tctccctcc
MA1653.1	ZNF148	8,76908	0,8280444	S100A10	128	139	+	tccgcctccctt
MA0079.3	SP1	3,72667	0,8280264	S100A10	53	63	+	attcctcctaa
MA1106.1	HIF1A	5,75451	0,8276157	S100A10	13	22	+	gtaggtgctc
MA0144.2	STAT3	2,48137	0,8276154	S100A10	94	104	-	ctctatggaaa
MA0481.3	FOXP1	6,77083	0,8272209	S100A10	89	99	-	tggaaaacaat
MA0712.2	OTX2	6,10042	0,8271849	S100A10	232	243	-	acaggataataa
MA0627.2	POU2F3	7,51878	0,8269195	S100A10	38	50	+	aaaatggaaaatt
MA0525.1	TP63	8,44859	0,8265241	S100A10	172	191	-	tctgatgcctatacatgccc
MA0079.3	SP1	3,57462	0,8261134	S100A10	330	340	-	gctccttctct
MA0620.3	MITF	7,85787	0,8252303	S100A10	340	357	-	cagaatgacatgactgtg
MA0620.3	MITF	7,85706	0,8252183	S100A10	340	357	+	cacagtcatgtcattctg
MA0764.2	ETV4	4,59132	0,8248294	S100A10	220	229	+	acagcaaggc
MA0148.4	FOXA1	7,58322	0,8245471	S100A10	412	423	-	gagtacacagga
MA1108.2	MXI1	3,19737	0,82378	S100A10	344	353	-	atgacatgac
MA0852.2	FOXK1	8,29999	0,8237752	S100A10	87	100	-	atggaaaacaattc
MA0442.2	SOX10	6,06158	0,8237646	S100A10	412	422	-	agtacacagga
MA0599.1	KLF5	1,59528	0,823547	S100A10	53	62	+	attcctccta
MA0508.3	PRDM1	6,64798	0,8235299	S100A10	100	110	-	agttttctcta
MA1115.1	POU5F1	7,05507	0,823244	S100A10	92	102	-	ctatggaaaac
MA0528.2	ZNF263	7,90948	0,8228508	S100A10	21	32	+	tcagggagggag
MA1116.1	RBPJ	4,74922	0,8227836	S100A10	92	101	-	tatggaaaac
MA0007.2	AR	5,97588	0,8227221	S100A10	418	432	-	cagtagatagagtac
MA0597.1	THAP1	5,03278	0,8216613	S100A10	39	47	-	tttccattt
MA0627.2	POU2F3	7,16997	0,8213475	S100A10	91	103	-	tctatggaaaaca
MA1596.1	ZNF460	11,4062	0,8210805	S100A10	125	140	+	gtctccgcctcccttc
MA0528.2	ZNF263	7,80969	0,8208787	S100A10	25	36	+	ggagggagaacc
MA1111.1	NR2F2	6,81054	0,8208055	S100A10	60	70	+	ctaaggtaact
MA0468.1	DUX4	1,37236	0,820637	S100A10	289	299	-	tcatcccatct
MA0037.2	GATA3	3,27945	0,8206011	S100A10	157	164	+	agacaaac
MA0627.2	POU2F3	7,11595	0,8204846	S100A10	108	120	+	actatgcagagaa
MA1579.1	ZBTB26	7,25893	0,8204789	S100A10	70	84	-	gactccacaaattta
MA1638.1	HAND2	3,0686	0,820322	S100A10	286	295	+	agaagatggg
MA1647.1	PRDM4	6,80954	0,8202963	S100A10	363	373	+	acctgtttagc

MA1647.1	PRDM4	6,78181	0,8196593	S100A10	146	156	+	acctgttgctc
MA0148.4	FOXA1	7,26974	0,8184414	S100A10	157	168	+	agacaaacatgt
MA1112.2	NR4A1	6,15799	0,8182224	S100A10	167	178	+	gtagagggcatg
MA1103.2	FOXK2	6,78695	0,818059	S100A10	89	99	-	tggaaaacaat
MA0481.3	FOXP1	6,301	0,8167703	S100A10	364	374	-	tgctaaacagg
MA1109.1	NEUROD1	6,83042	0,8165439	S100A10	285	297	+	tagaagatgggat
MA0600.1	RFX2	8,92774	0,8161716	S100A10	145	163	-	tttgtctgagcaacaggtc
MA0507.1	POU2F2	5,85036	0,8159873	S100A10	39	51	-	caattttccattt
MA0036.3	GATA2	5,48459	0,8158557	S100A10	232	242	+	ttattatcctg
MA0047.3	FOXA2	5,99001	0,8156263	S100A10	89	99	-	tggaaaacaat
MA0036.3	GATA2	5,47298	0,8156174	S100A10	328	338	-	tccttctcttc
MA0058.2	MAX	2,87399	0,8156128	S100A10	375	384	+	ggacacatat
MA0684.2	RUNX3	6,60503	0,8154138	S100A10	31	42	+	agaaccaaaaat
MA1115.1	POU5F1	6,70219	0,8154135	S100A10	163	173	+	acatgtagagg
MA0523.1	TCF7L2	5,27708	0,8153667	S100A10	266	279	+	gcactagaaaagca
MA0468.1	DUX4	0,88438	0,8153251	S100A10	66	76	-	aaatttagtta
MA1106.1	HIF1A	5,22862	0,8152331	S100A10	172	181	-	atacatgccc
MA0522.3	TCF3	5,72465	0,8151338	S100A10	316	326	-	aacacttgcta
MA0479.1	FOXH1	3,00158	0,8148516	S100A10	398	408	-	cacattccata
MA1601.1	ZNF75D	5,59602	0,8147141	S100A10	24	33	+	gggagggaga
MA1108.2	MXI1	2,71102	0,814692	S100A10	317	326	-	aacacttgct
MA1646.1	OSR2	7,20956	0,8146514	S100A10	424	435	-	gaacagtagata
MA0769.2	TCF7	5,90357	0,814059	S100A10	203	213	-	accttttattg
MA1683.1	FOXA3	6,41892	0,813724	S100A10	89	99	-	tggaaaacaat
MA0148.4	FOXA1	7,02546	0,8136835	S100A10	88	99	-	tggaaaacaatt
MA0852.2	FOXK1	7,7914	0,8135175	S100A10	362	375	-	ctgctaaacaggtc
MA1581.1	ZBTB6	7,44531	0,8134445	S100A10	16	28	-	ctccctgagcacc
MA0764.2	ETV4	3,94047	0,8134057	S100A10	21	30	+	tcagggaggg
MA0497.1	MEF2C	5,96521	0,8129639	S100A10	275	289	+	aagcaaaaagtagaa
MA1655.1	ZNF341	7,28578	0,8126583	S100A10	216	227	+	gtggacagcaag
MA0595.1	SREBF1	6,54012	0,8124924	S100A10	344	353	+	gtcatgtcat
MA1120.1	SOX13	5,87313	0,8123374	S100A10	85	95	-	aaacaattctg
MA0083.2	SRF	7,75033	0,8120381	S100A10	198	215	+	tacttcaataaaaggtga
MA1642.1	NEUROG2(var.2)	5,75586	0,8104431	S100A10	116	128	+	gagaaagatgtct
MA0597.1	THAP1	4,6594	0,8102341	S100A10	215	223	-	ctgtccact
MA0525.1	TP63	7,02279	0,8101645	S100A10	333	352	+	gaaggagcacagtcatgtca

MA1120.1	SOX13	5,75741	0,8096231	S100A10	437	447	-	caactatgaac
MA0510.1	RFX5	3,10376	0,8095652	S100A10	228	242	-	caggataataacagc
MA0830.2	TCF4	6,8197	0,8095618	S100A10	10	22	-	gagcacctacttt
MA1653.1	ZNF148	7,67264	0,8094854	S100A10	22	33	-	tctccctccctg
MA0782.2	PKNOX1	8,74699	0,8093717	S100A10	344	358	-	tcagaatgacatgac
MA1116.1	RBPJ	4,0894	0,8089917	S100A10	290	299	+	gatgggatga
MA0479.1	FOXH1	2,57453	0,8088441	S100A10	78	88	+	tggagtccaga
MA0047.3	FOXA2	5,64826	0,8088089	S100A10	422	432	-	cagtagataga
MA0052.4	MEF2A	8,27124	0,808683	S100A10	276	290	+	agcaaaaagtagaag
MA0745.2	SNAI2	5,64241	0,8085673	S100A10	142	154	+	taagacctgttgc
MA0809.2	TEAD4	6,29528	0,8085655	S100A10	49	60	+	ttgaattcctcc
MA1115.1	POU5F1	6,38031	0,8082708	S100A10	273	283	+	aaaagcaaaaa
MA0143.4	SOX2	5,53885	0,8081439	S100A10	425	435	-	gaacagtagat
MA0144.2	STAT3	0,872872	0,8081292	S100A10	188	198	+	cagattagaat
MA0468.1	DUX4	0,216561	0,8080556	S100A10	66	76	+	taactaaattt
MA1588.1	ZNF136	9,39566	0,8079884	S100A10	186	200	-	gtattctaatctgat
MA0047.3	FOXA2	5,60052	0,8078565	S100A10	63	73	+	aggtaactaaa
MA1648.1	TCF12(var.2)	5,86725	0,8075868	S100A10	316	326	-	aacacttgcta
MA0593.1	FOXP2	5,03596	0,8072891	S100A10	309	319	+	gagaaaatagc
MA0090.3	TEAD1	6,80235	0,8070529	S100A10	2	14	+	ggacattcaaagt
MA0501.1	MAF::NFE2	8,21556	0,8069284	S100A10	255	269	+	gtgagatagcagcac
MA0143.4	SOX2	5,45661	0,8063739	S100A10	106	116	+	aaactatgcag
MA0523.1	TCF7L2	4,61989	0,8062841	S100A10	3	16	+	gacattcaaagtag
MA0595.1	SREBF1	6,25698	0,806275	S100A10	291	300	-	ttcatcccat
MA1123.2	TWIST1	6,169	0,8062069	S100A10	116	128	+	gagaaagatgtct
MA0095.2	YY1	5,60002	0,8061252	S100A10	119	130	+	aaagatgtctcc
MA1651.1	ZFP42	10,6957	0,8060444	S100A10	115	135	+	agagaaagatgtctccgcctc
MA1102.2	CTCFL	6,45287	0,806022	S100A10	166	177	+	tgtagagggcat
MA0035.4	GATA1	5,718	0,805759	S100A10	328	338	-	tccttctcttc
MA0143.4	SOX2	5,41905	0,8055654	S100A10	437	447	-	caactatgaac
MA1119.1	SIX2	10,0303	0,8055497	S100A10	298	313	+	gaatgaaacacgagaa
MA0798.2	RFX3	8,08141	0,8052886	S100A10	148	161	+	ctgttgctcagaca
MA0468.1	DUX4	-0,0691959	0,804945	S100A10	297	307	+	tgaatgaaaca
MA1602.1	ZSCAN29	5,58063	0,8046652	S100A10	175	186	-	tgcctatacatg
MA0516.1	SP2	5,93889	0,8046323	S100A10	18	32	-	ctccctccctgagca
MA0039.4	KLF4	6,49423	0,8045273	S100A10	22	33	-	tctccctccctg

MA0035.4	GATA1	5,65714	0,8044474	S100A10	269	279	-	tgcttttctag
MA1116.1	RBPJ	3,86607	0,8043235	S100A10	40	49	+	aatggaaaat
MA0609.2	CREM	9,28191	0,8040147	S100A10	341	356	-	agaatgacatgactgt
MA0609.2	CREM	9,27963	0,8039706	S100A10	341	356	+	acagtcatgtcattct
MA1110.1	NR1H4	7,24486	0,803782	S100A10	346	356	-	agaatgacatg
MA0103.3	ZEB1	3,60927	0,803724	S100A10	206	216	-	ctcacctttta
MA1638.1	HAND2	2,1092	0,8036502	S100A10	183	192	-	atctgatgcc
MA0764.2	ETV4	3,36865	0,8033691	S100A10	315	324	+	atagcaagtg
MA0528.2	ZNF263	6,89305	0,8027636	S100A10	290	301	+	gatgggatgaat
MA0482.2	GATA4	6,61168	0,8027627	S100A10	113	124	-	atctttctctgc
MA0627.2	POU2F3	6,00172	0,8026855	S100A10	389	401	-	catacgcaaaagg
MA0627.2	POU2F3	5,98732	0,8024554	S100A10	189	201	-	agtattctaatct
MA1646.1	OSR2	6,65574	0,8016227	S100A10	408	419	-	acacaggagtgc
MA0036.3	GATA2	4,78667	0,8015323	S100A10	269	279	-	tgcttttctag
MA1602.1	ZSCAN29	5,36962	0,8013312	S100A10	124	135	+	tgtctccgcctc
MA0510.1	RFX5	2,3691	0,801189	S100A10	137	151	+	cttcttaagacctgt
MA0063.2	NKX2-5	5,2039	0,8010871	S100A10	265	275	+	agcactagaaa
MA0144.2	STAT3	0,267713	0,800798	S100A10	137	147	-	gtcttaagaag
MA0497.1	MEF2C	5,06466	0,8007969	S100A10	227	241	-	aggataataacagcc
MA0745.2	SNAI2	5,16819	0,800704	S100A10	359	371	+	ctagacctgttta
MA0083.2	SRF	6,79671	0,800557	S100A10	133	150	-	caggtcttaagaagggag
MA0144.2	STAT3	0,228613	0,8003243	S100A10	93	103	-	tctatggaaaa
MA0468.1	DUX4	-0,496846	0,8002898	S100A10	46	56	+	aaattgaattc
MA1647.1	PRDM4	5,93769	0,8002695	S100A10	428	438	+	tactgttccgt
MA0058.2	MAX	1,7746	0,8002065	S100A10	316	325	+	tagcaagtgt

References

1. Li, M.Z.1.; Elledge, S.J. SLIC: a method for sequence- and ligation-independent cloning. *Methods Mol. Biol.* **2012**, *852*, 51-59.

2. Pawlak, M..; Kikulska, A.; Wrzesinski, T.; Rausch, T.; Kwias, Z.; Wilczynski, B.; Benes, V.; Wesoly, J.; Wilanowski, T. Potential protective role of Grainyhead-like genes in the development of clear cell renal cell carcinoma. *Mol. Carcinog.* **2017**, *56*, 2414-2423
